# Efficacy and safety of traditional Chinese herbal medicine in the treatment of threatened abortion: A protocol for systematic review and meta-analysis

**DOI:** 10.1097/MD.0000000000023288

**Published:** 2021-02-05

**Authors:** Pengfei Zeng, Hang Zhou, Pei Guo, Wanting Xia, Jinzhu Huang, Qian Zeng

**Affiliations:** a Department of Gynecology, Hospital of Chengdu University of Traditional Chinese Medicine; b School of Nursing, Chengdu University of Traditional Chinese Medicine, Chengdu, Sichuan Province, China.

**Keywords:** protocol, systematic review, threatened abortion, traditional Chinese herbal medicine

## Abstract

**Background:**

Threatened abortion (TA) is the commonest complication that occurs in early pregnancy, especially in 8-12 gestational weeks when the secretion of estrogen and progesterone shifts from corpus luteum to placental. Conventional therapies are little evidence of their value. In China, traditional Chinese herbal medicine has been widely used for the treatment of TA for a long time. The lack of strong scientific evidences make this a priority area for research. We aim to evaluate the efficacy and safety of traditional Chinese herbal medicine in the treatment of TA, provide medical staffs with more useful information, and provide patients with better advises.

**Methods:**

We will search 8 databases and additional sources, including the Web of Science, PubMed, Cochrane Library, Embase, CBM, Wanfang, VIP, CNKI, and WHO ICTRP, ChiCTR, Clinical Trials, Grey Literature Database, for potentially eligible studies. Literature search, screening and retrieval are performed independently by two researchers. In the event of a dispute, a third party will be consulted to support the judgment. We will use RevmanV.5.3 to perform a fixed-effect meta-analysis for clinical homogeneity study data, and the level of evidence will be assessed using the GRADE method.

**Results:**

This systematic review and meta-analysis will put a high-quality synthesis of the efficacy and safety of traditional Chinese herbal medicine in the treatment of TA.

**Conclusion:**

The conclusion of this systematic review will provide evidence to assess traditional Chinese herbal medicine therapy whether is an efficacy and safe intervention to treat TA.

**Ethics and dissemination:**

Since this article does not contain patient personal information, ethical approval is not required. The contract is distributed by a peer-reviewed journal or conference report.

**Registration number:**

10.17605/OSF.IO/DG3T8

## 1 Introduction

Threatened abortion (TA), defined as vaginal bleeding with or without lower abdominal pain or backache with a closed cervix and an intrauterine viable fetus, is the commonest complication that occurs in early pregnancy, especially in 8 to 12 gestational weeks when the secretion of estrogen and progesterone shifts from corpus luteum to placental. It is the commonest complication in pregnancy, occurring in about a fifth of cases. And women with threatened abortion are 2.5 times more likely to miscarry than healthy women.^[1,2]^ As many as 50% of the miscarried fetuses and embryos have normal chromosomes.^[3]^ The recognized risk factors of TA are advanced age (maternal age over 35 years old), previous miscarriages, obesity, and cigarette smoking.^[2,4,5]^ In addition, uterine malformations, cervical incompetence, polycystic ovaries, poorly controlled diabetes mellitus, maternal infections, luteal phase defect, immune dysfunctions such as antiphospholipid syndrome, and exposure to environmental toxins also have association with TA.^[6–8]^ In clinical practice, targeted and directed treatment should be used to treat TA and prevent miscarriage when specific causes are identified, common drugs include uterine muscular relaxants,^[7]^ anti-D immunoglobulin,^[9]^ progesterone,^[10]^ β-HCG,^[11]^ magnesium sulfate, phloroglucinol,^[12]^ etc. However, in many cases, the cause of TA cannot be identified,^[13]^ treatment of TA is mostly empirical. Bed rest is routinely recommended, but there is insufficient evidence of high quality that supports it in order to prevent miscarriage.^[14]^

Since the knowledge of etiology and pathogenesis of TA is largely unclear, various interventions have been used in clinical practice, but the majority of them are still lack of sufficient evidence to support their use to prevent a miscarriage. Many parents prefer to seek alternative medicine. In China, traditional Chinese herbal medicine has been widely used for the treatment of TA for a long time.^[15]^ Although there is no scientific basis for the efficacy and safety claim, Chinese herbal medicine is commonly used to promote maternal and fetal health and to relieve medical problems during pregnancy worldwide. Compared with western medicine alone, a combination of Chinese herbal medicine and western medicine was more effective than western medicine alone for treating TA. No significant differences were found in adverse effects and toxicity, or in adverse pregnancy and perinatal outcomes.^[16]^ However, to our knowledge, the randomized controlled trials (RCTs) or systematic reviews (SRs) examining the efficacy and safety of traditional Chinese herbal medicine in the treatment of TA have never been systematically evaluated. The aim of this study was to access and review the available literature on the clinical applications of Chinese herbal medicine for TA, in order to provide scientific evidences and valuable references to clinical doctors and researchers for practices and studies.

## 2 Methods

### 2.1 Design and registration of the review

This SR has been registered on OSF and registration number is 10.17605/OSF.IO/DG3T8 and the protocol is based on the Preferred Reporting Items for Systematic Reviews and Metaanalyses, Protocols (PRISMA-P) guidelines.^[17]^

### 2.2 Inclusion criteria for study selection

#### 2.2.1 Type of study

All the studies of the efficacy and safety of traditional Chinese herbal medicine in the treatment of TA will be all RCTs without limitation on language or publication types restriction. Nonrandomized clinical studies, quasi-RCTs, cluster RCTs, and case reports will be excluded.

#### 2.2.2 Types of participants

Trials involving women with TA will be included.

#### 2.2.3 Types of interventions

Traditional Chinese herbal medicine and related treatments will be used in the intervention group. And control group will consist of drugs, placebo, or no intervention.

#### 2.2.4 Types of outcome

##### 2.2.4.1 Primary

The primary outcome was the incidence of miscarriage.

##### 2.2.4.2 Secondary

Secondary outcome included incidence of preterm birth in women without miscarriage (i.e., preterm delivery <37 weeks), neonatal mortality (defined as a death of a live-born baby within the first 28 days of life), and fetal genital abnormalities/virilization.

### 2.3 Data sources

We will search 8 databases and additional sources, including the Web of Science, PubMed, Cochrane Library, Embase, CBM, Wanfang, VIP, CNKI, and WHO ICTRP, ChiCTR, Clinical Trials, Grey Literature Database, for potentially eligible studies. RCTs which acupuncture on the efficacy and safety of traditional Chinese herbal medicine in the treatment of TA will be searched for independently by 2 reviewers in those sources.

### 2.4 Search strategy

The details are adjusted according to the specific sources including CBM, CNKI, WF, VIP, Web of Science, Embase, PubMed, Cochrane Library, WHO ICTRP, ChiCTR, Clinical Trials, and Grey Literature Database. The search strategy for PubMed is shown in Table 1.

**Table 1:** Search strategy for the PubMed database.

#1	threatened miscarriage [All Fields]
#2	threatened abortion [All Fields]
#3	#1 OR #2
#4	TCM [All Fields]
#5	Traditional Chinese Medicine [All Fields]
#6	Complement^∗^ medicine [All Fields]
#7	Alternative medicine [All Fields]
#8	#4 OR #5 OR #6 OR #7
#9	Chinese medicine[All Fields]
#10	Chinese herb^∗^[All Fields]
#11	Traditional Chinese Herbal Medicine[All Fields]
#12	herb^∗^ medicine[All Fields]
#13	herb[All Fields]
#14	#9 OR #10 OR #11 OR #12 OR #13
#15	#8 OR 14
#16	#3 AND #15
#17	clinical[All Fields]
#18	trial[All Fields]
#19	#17 AND #18
#20	clinical trials as topic[MeSH Terms]
#21	clinical trial[Publication Type]
#22	random^∗^[All Fields]
#23	random allocation[MeSH Terms]
#24	#19 OR #20 OR #21 OR #22 OR #23
#25	#16 AND #21

### 2.5 Data collection and analysis

#### 2.5.1 Selection of studies

**Figure 1:**
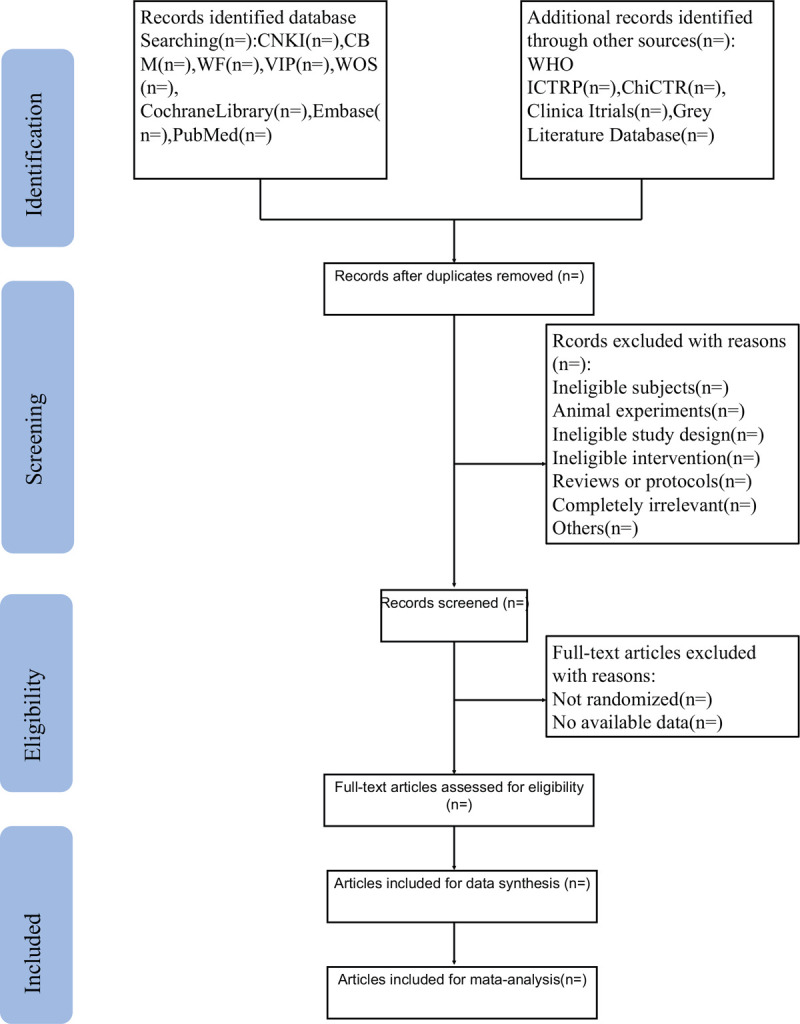
The Preferred Reporting Items for Systematic Reviews and Meta-analyses Protocols flow chart of selection process.

All reviewers will have a professional training about background, purpose, and process of the review. In the literature collection, the title and abstract of the literature will be 1st read to eliminate duplicate literature and the eligible studies searched will be uploaded to a database set up through NoteExpress. Two review authors will select and record independently through screening the titles, abstracts, and key words. Any disagreement about the inclusion of the studies will be resolved through discussion between the 2 review authors. If the discussion cannot reach an agreement, the arbiter will make a final decision of the study selection. If authors are similar or incidence data are extracted from the same database, the study period will be assessed. Details of the selection procedure for studies are shown in a PRISMA-P flow chart (Fig. 1).

#### 2.5.2 Data extraction and management

Data extraction will be also conducted by 2 researchers independently through a standardized eligibility form. In case of disagreement, a 3rd party (the arbiter) shall be consulted to assist judgment, and the missing information shall be supplemented by contacting the author. The general information of the selected articles will be extracted, including 1st author, year of publication, country, study design, sample size, detailed intervention, control treatment, duration of disease, duration of follow-up, and the like. When the data of articles are insufficient or ambiguous, one of the authors will in contact with the original author to request detailed about the research by e-mail or telephone or estimate the data.

#### 2.5.3 Assessment of risk bias

Two review authors will measure the risk of bias of the included studies with Cochrane Handbook V.5.3.0 independently, which includes the following 7 items: sequence generation, blinding of participants, blinding of outcome assessors, allocation concealment, incomplete outcome data, selective outcome reporting, and other sources of bias. It will be ranked risk level within categorized as low risk of bias, unclear risk of bias, and high risk of bias. In case of disagreement, the arbiter shall be consulted to assist judgment.

#### 2.5.4 Measures of treatment effect

The dichotomous data will be analyzed by relative risk (RR) ratio with 95% confidence intervals (CIs), and the mean difference (MD) or standard MD (SMD) with 95% CIs will be used to estimate the continuous data.

#### 2.5.5 Management of missing data

Where possible, we will analyze the data according to the intention-to-treat. If there is missing or incomplete data, we will contact the original investigator to verify the study characteristics and obtain missing numerical result data. If the missing data are not available, then this analysis will depend on the data available.

#### 2.5.6 Assessment of heterogeneity

According to the Cochrane Handbook, we will choose the *I*^2^ statistic to measure heterogeneity among the studies in every analysis. When *P* > .1, *I*^2^ < 50%, it is considered that there is no heterogeneity between the experiment, and the fixed effects model will be used for statistics, otherwise, the random effects model is adopted to analyze.

#### 2.5.7 Assessment of reporting biases

If the number of included studies is >10, we will use funnel plots to measure publication bias. If funnel chart is evenly distributed, it indicates no reporting bias, and vice versa.

#### 2.5.8 Data synthesis

The data will be analyzed and synthesized through Review Manager 5.3 software which from Cochrane Collaboration will be employed to compute the data synthesis. The fixed effects model (*I*^2^ < 50%) or random effects model (*I*^2^ ≥ 50%) will be selected. All data will be analyzed with 95% CIs. The dichotomous data will be analyzed by RR, and the continuous data will be analyzed by MD or SMD.

#### 2.5.9 Subgroup analysis

If we find substantial heterogeneity, subgroup analysis will be implemented according to acupuncture types, outcome measures, and the like.

#### 2.5.10 Sensitivity analysis

We will carry out sensitivity analysis to identify the quality and robustness of the results in the review. The principal criteria include methodological quality, sample size, and analysis issue (such as missing data's efficacy). The meta-analysis will be operated repeatedly.

#### 2.5.11 Grading the quality of evidence

The reviewers will use the GRADE rating standards.^[18]^ The GRADE system will be used to GRADE the obtained outcome indicators from 5 items of research limitations, inconsistency, indirectness, inaccuracy, and publication bias. In the case of the RCTs, the GRADE classifies the evidence of the outcome indicators evaluated by the system, and all the outcome indicators are graded by quality through the GRADE rating standards. Then, evidence quality will be rated “high,” “moderate,” “low,” or “very low” according to the GRADE rating standards. The quality of the evidence is high, indicating that future research is unlikely to change existing evidence; “moderate” indicates that future research may have an important impact on existing evidence, and may change the evaluation results; being low-level indicates that future research is likely to have a significant impact on existing evidence and may change the evaluation results; “very low” indicating that all existing evidence is highly uncertain.

## 3 Publication plan

The systematic review will be published in peer-reviewed journals in both electronic and print versions.

## 4 Discussion

TA is a common complication of pregnancy occurring in 15% to 20% of all clinically recognized pregnancies.^[19]^ Bed rest and using progestogens are conventionally the most commonly used management technique for threatened miscarriage. However, there is little evidence of their value.^[14,17]^

In China, women with TA will be treated with traditional Chinese herbal medicine to try and decrease the risk of miscarriage. The evidence for the effectiveness of this treatment has been inconclusive, but data from a meta-analysis suggest that a combination of Chinese herbal medicine and western medicine was more effective than western medicine alone.^[20]^ However, there are theoretical risks to prescribing any treatment in pregnancy and for many practitioners this will be a major change in practice. Due to limited efficacy and safety information on these herbs,^[21–23]^ making this is a priority area for research.

We expect that this SR will provide strong evidence for the efficacy and safety of current traditional Chinese herbal medicine in the treatment of TA, provide medical staffs with more useful information, and provide patients with better advises. There may be some limitations in this SR, including language limitations, lack of research, and the like, which may lead to substantial heterogeneity.

## Author contributions

**Conceptualization:** Pengfei Zeng, Hang Zhou, Qian Zeng.

**Data curation:** Pengfei Zeng, Pei Guo.

**Formal analysis:** Hang Zhou, Wanting Xia, Jinzhu Huang.

**Funding acquisition:** Qian Zeng.

**Methodology:** Pengfei Zeng, Wanting Xia, Jinzhu Huang.

**Project administration:** Qian Zeng.

**Supervision:** Pei Guo.

**Writing – original draft:** Pengfei Zeng, Hang Zhou.

**Writing – review & editing:** Pengfei Zeng, Hang Zhou.

## References

[1] Sotiriadis A Papatheodorou S Makrydimas G Threatened miscarriage: evaluation and management. BMJ 2004;329:152–5. 15258071 10.1136/bmj.329.7458.152 PMC478228

[2] Makrydimas G Sebire NJ Lolis D Fetal loss following ultrasound diagnosis of a live fetus at 6-10 weeks of gestation. Ultrasound Obstet Gynecol 2003;22:368–72. 14528471 10.1002/uog.204

[3] Suzumori N Sugiura-Ogasawara M Genetic factors as a cause of miscarriage. Curr Med Chem 2010;17:3431–7. 20712563 10.2174/092986710793176302

[4] Falco P Milano V Pilu G Sonography of pregnancies with first-trimester bleeding and a viable embryo: a study of prognostic indicators by logistic regression analysis. Ultrasound Obstet Gynecol 1996;7:165–9. 8705406 10.1046/j.1469-0705.1996.07030165.x

[5] Hahn KA Hatch EE Rothman KJ Body size and risk of spontaneous abortion among danish pregnancy planners. Paediatr Perinat Epidemiol 2014;28:412–23. 25225009 10.1111/ppe.12142 PMC4356022

[6] Tien JC Tan TY Non-surgical interventions for threatened and recurrent miscarriages. Singapore Med J 2007;48:1074–90. quiz 1090. 18043834

[7] Lede R Duley L Uterine muscle relaxant drugs for threatened miscarriage. Cochrane Database Syst Rev 2005;3:CD002857. 10.1002/14651858.CD002857.pub2 PMC8453480 16034877

[8] Xu Q Chen J Wei Z Sex Hormone Metabolism and Threatened Abortion. Med Sci Monit 2017;23:5041–8. 29056745 10.12659/MSM.904500 PMC5665605

[9] Weinberg L Use of anti-D immunoglobulin in the treatment of threatened miscarriage in the accident and emergency department. Emerg Med J 2001;18:444–7. 11696491 10.1136/emj.18.6.444 PMC1725734

[10] Wahabi HA Fayed AA Esmaeil SA Progestogen for treating threatened miscarriage. Cochrane Database Syst Rev 2018;8:CD005943 Published 2018 Aug 6. 30081430 10.1002/14651858.CD005943.pub5 PMC6513446

[11] Qureshi NS Treatment options for threatened miscarriage. Maturitas 2009;65: Suppl 1: S35–41. 19945236 10.1016/j.maturitas.2009.10.010

[12] Yuan S Gao F Xin Z Comparison of the efficacy and safety of phloroglucinol and magnesium sulfate in the treatment of threatened abortion: A meta-analysis of randomized controlled trials. Medicine (Baltimore) 2019;98:e16026. 31192955 10.1097/MD.0000000000016026 PMC6587576

[13] Stray-Pedersen B Stray-Pedersen S Etiologic factors and subsequent reproductive performance in 195 couples with a prior history of habitual abortion. Am J Obstet Gynecol 1984;148:140–6. 6691389 10.1016/s0002-9378(84)80164-7

[14] Aleman A Althabe F Belizán J Bed rest during pregnancy for preventing miscarriage. Cochrane Database Syst Rev 2005;2:CD003576. 10.1002/14651858.CD003576.pub2 PMC8721648 15846669

[15] Giovanni M The foundations of Chinese medicine: a comprehensive text for acupuncturists and herbalists [J]. 1989;Churchill Livingstone, Edinburgh, UK: 219-268.

[16] Li L Dou LX Neilson JP Adverse outcomes of Chinese medicines used for threatened miscarriage: a systematic review and meta-analysis. Hum Reprod Update 2012;18:504–24. 22661551 10.1093/humupd/dms025

[17] Shamseer L Moher D Clarke M Preferred reporting items for systematic review and meta-analysis protocols (PRISMA-P) 2015: elaboration and explanation. BMJ 2015;350:g7647. 25555855 10.1136/bmj.g7647

[18] Guyatt GH Oxman AD Schünemann HJ GRADE guidelines: a new series of articles in the Journal of Clinical Epidemiology. J Clin Epidemiol 2011;64:380–2. 21185693 10.1016/j.jclinepi.2010.09.011

[19] National Collaborating Centre for Women's and Children's Health (UK). Ectopic Pregnancy and Miscarriage: Diagnosis and Initial Management in Early Pregnancy of Ectopic Pregnancy and Miscarriage. London: RCOG; December 2012. 23638497

[20] Wahabi HA Fayed AA Esmaeil SA Progestogen for treating threatened miscarriage. Cochrane Database Syst Rev 2011;CD005943 Published 2011 Dec 7. 10.1002/14651858.CD005943.pub4 22161393

[21] Chuang CH Chang PJ Hsieh WS Chinese herbal medicine use in Taiwan during pregnancy and the postpartum period: a population-based cohort study. Int J Nurs Stud 2009;46:787–95. 19193377 10.1016/j.ijnurstu.2008.12.015

[22] Wiebrecht A Gaus W Becker S Safety aspects of Chinese herbal medicine in pregnancy-re-evaluation of experimental data of two animal studies and the clinical experience. Complement Ther Med 2014;22:954–64. 25440387 10.1016/j.ctim.2014.08.005

[23] Li L Tang LY Man GC Potential reproductive toxicity of Largehead Atractylodes Rhizome, the most commonly used Chinese medicine for threatened miscarriage. Hum Reprod 2011;26:3280–8. 21984574 10.1093/humrep/der335

